# Self-rated health and venous thromboembolism among middle-aged women: a population-based cohort study

**DOI:** 10.1007/s11239-019-01995-7

**Published:** 2019-11-19

**Authors:** Peter Nymberg, Emelie Stenman, Susanna Calling, Jan Sundquist, Kristina Sundquist, Bengt Zöller

**Affiliations:** Region Skåne, Center for Primary Health Care Research, Jan Waldenströms gata 35, Skåne University Hospital Malmö, University Hospital, Lund University, Jan Waldenströms gata 35, 205 02 Malmö, Sweden

**Keywords:** Venous thromboembolism, Varicose veins, Prevention, Self-rated health, Women

## Abstract

Venous thromboembolism (VTE) is one of the most common types of cardiovascular diseases (CVDs) and is associated with increased mortality-risk. Poor-self rated health (SHR) has been associated with elevated inflammatory markers and CVDs. However, little is known about as a predictor of incident VTE. To examine the association between self-rated health, lifestyle and incident VTE among middle-aged women. 6917 women aged 50–64 years, followed for 20 years in the Women’s Health In the Lund Area (WHILA) study. After exclusion of those who medicated with anticoagulants, were living in nursing homes or suffered from cancer, stroke, VTE or CHD before baseline, a cohort of 5626 women remained. Cox regression was used to analyse the relationship between self-rated health and time to VTE, censored for any of the previous mentioned diseases during follow-up. Data were collected by questionnaires, physical examinations and Swedish registers. In total, 220 women were affected by VTE corresponding to an incidence rate of 3.9 per 1000 person-years. Adjustment for self-rated health did not significantly predict incident VTE, and neither did any of the lifestyle-related habits (e.g. physical activity and dietary habits including alcohol consumption), besides smoking. This study supports previous results with varicose veins and waist circumference as strong predictors of VTE. Poor self-rated health does not seem to be a valid predictor of VTE. Among lifestyle-related parameters, smoking was significantly associated with risk of VTE. We could also confirm the effect of the other already known risk factors.

**Electronic supplementary material:**

The online version of this article (10.1007/s11239-019-01995-7) contains supplementary material, which is available to authorized users.

## Highlights

- Poor self-rated health does not seem to be useful as a predictor of VTE.
- Varicose veins, smoking and waist circumference showed a association with risk of incident VTE.
- Other lifestyle associated behaviors do not seem to have any strong effect on risk of incident VTE.
- Prevention in those with varicose veins can be important.

## Introduction

The incidence of venous thromboembolism (VTE) exceeds 1 per 1000 persons-years. More than 200,000 new cases of VTE occur in the United States each year, with a corresponding mortality rate of about 30% within 30 days [1]. Among the survivors, 30% develop recurrent VTE [1] and there is an increased mortality risk up to 8 years after the first thrombosis, [1, 2]. Provoked VTE occurs in relation to identifiable risk factors, such as pregnancy, bone fractures, or surgery [1, 3]. Unprovoked VTE shares some known risk factors with other cardiovascular diseases (CVDs) such as obesity, smoking [4–7] and sleep-apnoea [8–10]. Other risk factors for VTE include high body height [11] and varicose veins [1, 12–15]. Varicose veins and VTE has been suggested to share familial susceptibility [16] and a genetic component of the familial clustering has been found for VTE, which makes heredity a potent risk factor [17, 18] The risk of VTE also increases with age, especially among men aged > 50 years [19, 20].

 Poor self-rated health (SRH) has been associated with elevated serum inflammatory markers [21] and has also been shown to be a predictor of depression [22], stroke [23] and other CVDs [24], diabetes [25], lung-cancer [26] and all-cause mortality [27, 28]. Thus, SRH is usually considered a valid and efficient measure of mental and physical health, especially in women [29], although there is still a need to identify the validity limits of SRH for prediction of illness and mortality [30].

A previous study investigating associations between the first VTE-episode and work-related disability showed a reduced risk for VTE when adjusted for good SRH. The authors discussed a possible association between low SRH and an elevated risk of incident VTE [31].

To the authors’ knowledge, there are no previously published studies regarding the longitudinal association between SRH and incident VTE later in life among middle aged women. If such an association exists, it can be useful to include in risk assessment for incident VTE. In addition, as women often are more affected by poor SRH, they are particularly suitable for a study of a possible longitudinal association with VTE. SRH is also better at predicting somatic diseases in women than in men [29].

The main aim of this cohort study was to examine the association between baseline SRH in middle-aged women (50–64 years) and incident VTE. The second aim was to analyze the association between lifestyle habits, i.e. physical activity, diet, smoking, alcohol consumption and VTE.

## Method

### Study population

The Women´s Health In the Lund Area (WHILA) is a prospective cohort study in southern Sweden. Sample characteristics, data collection and clinical definitions for WHILA have been described previously [32]. Briefly, the study invited all women (n = 10,766) living in any of the five municipalities around the city of Lund by December 1995, and who were born between December 1935 and December 1945, to a screening procedure that took place from December 1995 until February 2000. A total of 6917 women consented to participate in the study, of which 6916 had complete datasets. The study population was identified through a population registry that comprised all inhabitants. A health-screening program included laboratory examinations, blood samples and a basic baseline questionnaire that was mailed along with the invitation and collected in conjunction with the first examination. The baseline questionnaire included 104 questions concerning education, household, working status, perimenopausal status, medical history, drug treatment, personal and family history of diabetes or cardiovascular disease (myocardial infarction, stroke, deep venous thrombosis or hypertension in parents or siblings with an event before the age of 60 years). It also contained questions about habits like smoking, alcohol consumption, physical activity, general dietary habits, quality of life, as well as subjective physical and mental symptoms. This questionnaire was a composite of several pre-existing and validated questionnaires.

### Measurements and definitions

Information about diseases and medication was obtained from the Swedish Hospital Discharge Register, the Hospital Outpatient Register and the Swedish Cancer Register in addition to self-reported data from WHILA study baseline questionnaires and measurements. The definition of VTE includes the following ICD-codes; ICD-7; 463-466, 583.00 334.40, 334,50 682, 684. ICD-8 321, 450-453, 671 (not 671,00) 673,9. ICD-9; 437G, 451-453, 415B, 416 W, 671C, D, E, F, X, 673C, 639G. ICD-10; I26, I636, I676, I80-82, O222-225, O229, O870-873, O879, O882, O082, O087. We excluded from our analyses participants with stroke (ICD-7; 331, 332, 334.09. ICD-8; 430-434, 436. ICD-9; 430-434, 436. ICD-10; I60-I64 (not I63.6).) or CVD (ICD-7; 420. ICD-8; 410-414. ICD-9; 410-414. ICD-10; I20-I25.), cancer (ICD-7; 140–209) or VTE before baseline, both self-reported and by register. In addition, we excluded women who were living in nursing homes or similar at baseline or medicated with warfarin, dicumarol, or antithrombin (ATC-code; B01AA03, B01AA01, and B01AB02). After exclusion, 5626 women remained (Table 1).

**Table 1:** Number of exclusions and reason for exclusion both by register and self-reported

Total included	6916
No. of living in nursing homes or similar	190
No. of prevalent VTE diagnoses	321
No. of prevalent CHD	104
No. of prevalent stroke	79
No. of prevalent cancer	716
No. of anticoagulant (not ASA)	30
Remaining after exclusion	5626

The same person can occur in several groups

 SRH was assessed only at baseline from a single question, in which the participants were asked to rate their perceived health in a 7-graded Likert scale from “Very poor” to “Excellent, could not be better” (1 = very poor, 7 = Excellent). In this study, we classified alternative 1–4 as poor SRH and 5–7 as good SRH. Weight and height were rounded off to the nearest 0.1 kg and 0.5 cm. Body mass index (BMI) was calculated as weight in kilograms divided by height in meters squared (kg/m^2^). BMI was considered as underweight if BMI was < 18.5, normal weight if BMI = 18.5–24.9, overweight if BMI = 25.0–29.9, obese class I if BMI = 30.0–34.9, obese class II if BMI = 35.0–39.9 and obese class III if BMI was > 39.9. BMI class II and class III were put together due to small groups. Subjects were categorized as current smokers (i.e. those who smoked regularly or occasionally) or non-smokers (i.e. former smokers and never smokers). Low physical activity, i.e. less than 30 min vigorous activity 5 days a week [33] was defined as the lower tertile among the answering alternatives (i.e. very low and low) (Table 2).

**Table 2:** Definition of activity groups

Very-high	Hard training several times per week
High	> 3 h of running, skiing swimming
Middle-high	1–2 h/week with running, swimming, gymnastics, 4 h light physical activity/week and total responsibility of household tasks
Middle	Light activity 2–4 h/week walking, gardening dancing. Head of responsibility of household tasks
Low	Mostly sitting, light household work or gardening but not head of responsibility of the tasks
Very low	Hardly any physical activity at all

 Diet was defined by questions about intake of fat, sugar, fruit or dietary fiber. If high intake of sugar or fat or low intake of dietary fiber or fruit were reported, we considered the diet as less healthy [32]. If low intake of sugar and fat and high intake of fruit and dietary fiber was reported, we considered the diet as healthy. Education was categorized into three classes; comprehensive school (9 years), upper secondary school (12 years), and university degree. In the multivariate models model, we used waist circumference instead of BMI; this was due to previous studies showing waist circumference as a valid predictor for VTE [34].

### Statistical analysis

P-values were calculated with two-sided Student’s *t* test for continuous variables and with χ^2^-test for dichotomized variables. Cox proportional hazards regression was used to analyze the relationship between SRH and time to VTE. Hazard ratios (HR) with 95% confidence intervals (CI) were calculated. Those who were diagnosed with hypertension or varicose veins after baseline were censored, as well as those who were affected by stroke, CHD or cancer diagnosis before the VTE during the follow-up time. In the multivariate model, only confounding variables that showed a significantly increased incident VTE-risk in the univariate test were included. In the first model, we adjusted for age. In the second model, we added physical activity, smoking, and waist circumference. In the third model, we completed with varicose veins and hypertension. The proportional hazard assumption was tested with Schoenfeld residuals [35, 36]. Analyses were performed in STATA 14.1.

To test the robustness of the results, we investigated the cohort in different ways, at first without excluding prevalent disease (VTE, CHD, stroke, cancer) before baseline. At the next step, we excluded all the patients both prevalent (VTE, CHD, stroke, cancer) and censured incident (CHD, stroke cancer) before VTE. We got almost the same result regardless of which we excluded, there were only small differences in hazard ratios and levels of significance. When the proportional hazard assumption test was significant in the two first models thus meaning the effect were not constant and changed over the follow-up time. We investigated the material by breaking it up into shorter time span of 5 years (0–5, 5–10, 10–15, 15–20). All the different time spans showed proportionality except for the time span 0-5 years. When excluding only the 0–5 year to get broader time span, there was proportionality in all models between 5 and 20 years follow up.

## Results

 During a follow-up time of 20.4 years, a total of 220 women were affected by VTE. The sum of the follow-up time was 85,645.836 years corresponding to a VTE incidence rate of 3.9 (95% CI 2.26–2.94) per 1000 person-years. There appeared to be statistically significant differences between women that were affected by incident VTE versus women that were not affected, i.e. differences in weight, height, waist-hip ratio, smoking, high and low activity level, dietary fiber, overall diet, SRH and varicose veins.

 Women with incident VTE were on average taller, heavier and had a greater waist-hip ratio than those without incident VTE. There was a higher percentage of smokers and women with a low physical active level in the group with incident VTE. Regarding diet, there were only significant differences between the groups in dietary fiber intake and overall diet. Among women with incident VTE, a higher percentage had a low intake of dietary fiber and a less healthy overall diet. In the incident VTE-group there was also a greater number with varicose veins. The SRH differed significantly between the groups with a higher percentage rating their health as poor in the incident VTE group and this was even more obvious when categorizing into poor or good SRH.

An unadjusted Cox regression analysis was conducted (Table 3) with the aim to investigate if there were any significant associations between the variables that fell out with significance in the former analysis and incident VTE.

**Table 3:** Univariate Cox regression with those variables which differed with a statistical significance in the baseline characteristics

	Hazard ratio	95% CI	p	n	Failures
Age	1.05	1.00–1.10	.033	5626	220
Marital				5606	220
Unmarried	1.22	.75–1.99	.42		
Married	Ref				
Divorced	.99	.68–1.44	.961		
Widowed	1.10	.60–2.03	.757		
Education				5523	214
7–9 years	1.42	.99–2.00	.052		
10–12 years	Ref				
> 12 years	1.18	.86–1.63	.294		
Physical activity				5539	216
Very low	2.27	.92–5.59	.075		
Low	1.56	.88–2.74	.125		
Middle	Ref				
Middle-high	1.02	.76–1.35	.914		
High	.76	.33–1.73	.508		
Very high	6.34E–16	0	1.000		
Activity high/low	1.70	1.07–2.74	.026	5539	216
Current smoker	1.43	1.04–1.96	.028	5533	216
Former smoker	.92	.65–1.32	.656		
(Ref non-smoker)					
Height	1.03	1.00–1.06	.013	5482	213
Waist circumference	1.03	1.02–1.04	0	5041	198
Alcohol				5398	211
0g/w	1.26	.93–1.70	.135		
0–12g/w	Ref				
> 12g/w < 130g/w	.77	.48–1.22	.265		
Sugar				5580	216
Daily	1.70	.97–3.01	.066		
Sometimes	Ref				
Avoids	1.05	.78–1.42	.748		
Fat in food				5353	205
Much	1.46	.89–2.40	.132		
Careful with	Ref				
Avoids	1.11	.82–1.49	.509		
Fiber				5547	215
Low intake	1.16	.42–3.13	.771		
Regularly	Ref				
Much	.77	.58–1.03	.078		
Fruit				5594	219
Rarely	1	.76–1.32	.985		
Regularly	Ref				
Much	.76	.24–2.41	.644		
Portion size				5240	198
Big	.81	.45–1.46	.481		
Regular	Ref				
Small	.95	.70–1.30	.753		
Diet	1.38	.94–2.00	.095	5612	220
Family history				5477	216
Yes	1.38	.93–2.06	.112		
No	Ref				
Don’t know	1.11	.68–1.83	.675		
Acetylsalicylic	1.52	.38–6.12	.554	5626	221
Diabetes	.62	.28–1.41	.261	5577	221
Hypertension	1.17	.67–2.05	.581	5626	220
Varicose veins	2.7	1.47–4.95	.001	5626	220
Self-rated health				5529	216
1. Very poor	2.78	.99–7.80	.051		
2.	.71	.25–1.99	.516		
3.	1.15	.66–2.01	.628		
4.	Ref				
5.	.83	.55–1.25	.374		
6.	.7	.47–1.05	.087		
7. Excellent	.54	.34–.85	.008		
Poor self-rated health	1.51	1.13–2.03	.005	5529	216

Self-rated health was dichotomized into the variable Poor self-rated health, with group 1–4 as poor self-rated health and 5–7 as good self-rated health

Despite a relatively small difference in mean age between the groups at baseline, there was an increased risk for incident VTE for every year older at baseline (HR 1.05, 95% CI 1.00–1.10, p = 0.033). Cox regression analysis of SRH did not show any significant association with VTE-risk except for a decreased risk for those who rated their health as excellent (HR 0.54, CI 0.34–0.85, p = 0.008). When dichotomizing SRH into poor and good respectively, there was an increased risk for those who rated their health as poor (hazard ratio 1.51, CI 1.13–2.03, p = 0.005). When we dichotomized the variable physical activity, there was an increased incident VTE-risk for the low group (HR 1.70, CI 1.07–2.74, p = 0.026). Smoking at baseline was associated with a 43% increased risk of incident VTE (HR 1.43, CI 1.04–1.96, p = 0.028), smokers who had quit smoking at least one month before inclusion had a non-significant decreased risk of incident VTE (0.92, 0.65–1.32, p = 656). Waist circumference and varicose veins were associated with an increased risk as well (HR 1.03, CI 1.02–1.04, p = 0.000 and HR 2.70, CI 2.47–4.95, p = 0.001 respectively). Knowledge about family history of VTE (parents or siblings) was not associated with increased incident VTE-risk (HR 1.38, CI 0.93–2.06, p = 0.112). There was a non-significant trend towards an association between unhealthy diet and risk of incident VTE (HR 1.38, CI 0.44–2.00, p = 0.095). There was no significant association between hypertension before baseline and incident VTE (HR1.17, CI. 0.67–2.05, p = 0.581).

When the Cox regression results were adjusted for age (Table 4), model 1 showed a 51% increased risk for incident VTE if SRH was poor (HR 1.51, CI 1.12-2.02 p = 0.006). When we adjusted for lifestyle-related variables; physical activity, smoking, former smoking and waist circumference in model 2, the association between poor SRH and increased risk of incident VTE decreased to 16% and was not significant (HR 1.16, CI 0.84–1.61 p = 0.370). Even if low physical activity was associated with a significantly increased risk of incident VTE in the unadjusted model, this association was reduced and not significant when adjusting for other variables (HR 1.19, CI 0.62–2.04, p = 0.526). In model 3, adjusting for varicose veins and hypertension, the association between SRH and risk of incident VTE increased to 18% (HR 1.18, CI 0.85–1.65 p = 0.315). Even though the increased risk of age remained, it was not statistically significant in any of the models. In model 3, only the well-known risk factors had significant effect; smoking (HR 1.44, CI 1.02–2.03, p = 0.037), waist circumference (HR 1.03, CI 1.02–1.04, p = 0.000) and varicose veins (HR 2.60 CI 1.40–4.80, p = 0.002). When testing the proportional hazards assumptions for model 1, the global test was significant (p = 0.0390), which means that the hazards were not proportional over time. The second and third model showed proportional hazards (p = 0.1290, p = 0.1872) in the global test.

**Table 4:** Multivariate Cox regression with the confounding variables that showed a significantly increased incident VTE-risk in the univariate test (Table 3)

Total (n)	5529	4806	4806
Failures (n)	216	188	188
Self-rated health (poor/good)	1.51	1.16	1.18
CI	1.12–2.02	.84–1.61	.85–1.65
p	.006	.37	.315
Age	1.05	1.03	1.03
CI	1.00–1.09	.98–1.08	.98–1.08
p	.048	.193	.200
Physical activity (low/high)		1.19	.88
CI		.69–2.04	.65–1.19
p		.526	.421
Current smoker*		1.44	1.44
CI		1.02–2.03	1.02–2.03
p		.037	.037
Former smoker*		.89	.90
CI		.61–1.30	.62–1.32
p		.549	.588
Waist circumference		1.03	1.03
CI		1.02–1.04	1.02–1.04
p		0	0
Varicose veins (yes/no)			2.6
CI			1.40–4.80
p			.002
Hypertension (yes/no)			1.12
CI			.63–1.98
p			.696
Proportional hazard assumption test	0.039	0.1290	0.1872

1st model adjusted by age and SRH. 2nd model adjusted by model 1, dichotomized level of physical activity, smoking former smoker and waist circumference. Third model is adjusted by model 2, varicose veins and hypertension

*Reference is non-smoker

## Discussion

This is, to our knowledge, the first study that examines SRH as a predictor of incident VTE among middle-aged women in a well-defined cohort. In the multivariate model 3, there was a tendency towards an association between poor SRH and risk of incident VTE, but it was not statistically significant. Our results confirmed, however, an association with the already known risk factors varicose veins, smoking and waist circumference [4, 11, 12, 34].

Braekkan et al. suggested that good SRH could attenuate the risk of VTE among people with a permanent work-related disability. They discussed the possibility that poor SRH may be affected by other diseases that in turn may increase the risk of VTE [31]. If poor SRH was a good predictor of incident VTE, we ought to have observed a significant association in this cohort comprising only women. However, we did not observe any significant association. The reason for this assumption is that SRH has been suggested to be better at predicting different diseases in women than in men [29]. What is notable, however, is that when we excluded those who got affected with VTE between baseline and 5 years follow up, the risk for VTE among those with poor SRH was increased even in the fully adjusted model (HR 1.38, CI 0.96–1.99, p = 0.086). This shows that there may be a significant association during long time follow-up, but we were unable to capture it in this study. Regarding the association between varicose veins and VTE, Chang et al. [15] pointed out, that it is unknown whether this association is causal or represents a common set of risk factors. Considering the strong association, it can be argued for the need of preventive actions in people with varicose veins. This is especially pertinent since about 40% of adults are affected by varicose veins, and even a higher share among those who are obese and women with more than two pregnancies [37].

Low physical activity, i.e. less than 30 min vigorous activity 5 days a week [33], was significantly associated with increased risk of incident VTE in the unadjusted model, but not in the multivariate model 3 (Table 4). This may have been due to lack of power. There were no significant associations between self-reported intake of healthy or unhealthy food, alcohol, portion size and risk of incident VTE, which is consistent with previous studies among women, even if there have been suggested associations between unhealthy food, activity level and overweight/obesity [38, 39]. We neither noticed any significant associations between hypertension and risk for VTE, or differences between those who got affected with incident VTE and those who did not, regarding hypertension. Healthy food and a proper amount of physical activity do not seem to prevent VTE, but a healthy way of living may help to prevent a large waist circumference, which turned out to be associated with incident VTE in our study.

Why SRH is associated with arterial CVDs [24] but not with VTE in the present study may have several explanations. It is possible that the study participants changed their way of living after baseline measurements including questionnaires if they became more aware of their negative habits and changed them [40, 41]. Another explanation could be that many risk factors are different between VTE and arterial diseases [7], although some risk factors are shared. It is thus possible that SRH is a risk factor for arterial diseases but not for VTE. SRH may also be a weak risk factor for VTE and that a larger study might find a significant association with SRH, albeit weaker than for arterial disease. Finally, it is possible that SRH does not represent an additional risk factor for VTE once the other risk factors have been taken into account.

### Strengths

This study is comprised of a well-defined cohort and it contained both self-reported and anthropometric values combined with information from registers. We censored participants who were affected by cancer or any cardiovascular disease before the first VTE occurrence during follow-up and diagnosed hypertension and varicose veins after baseline in order to decrease the risk of influences on VTE-risk and poor SRH.

### Limitations

A limitation is that SRH was only measured at baseline as SRH may change over time. However, a study by Sargent-Cox et al. reported that SRH in women remained relatively stable compared with increasing age, whereas men´s ratings tended to become more negative [42]. The study was performed in middle-aged women living in a certain area, which limits the generalizability to a wider context, e.g. to men and women in other ages than those enrolled. Due to a limited number of VTE events, we could not compare the occurrence of the different VTE-forms deep vein thrombosis (DVT) and pulmonary embolism (PE) between the groups. For the same reason, we could not perform any sub-analysis with provoked and unprovoked VTE.

## Conclusions

 Poor self-rated health does not seem to be useful as a predictor of VTE. Lifestyle associated behaviors such as diet, alcohol consumption and physical activity or hypertension do not seem to have any strong effect on risk of incident VTE either. On the other hand, we could confirm the effect of already known risk factors; varicose veins, smoking and waist circumference. Varicose veins showed a strong association with risk of incident VTE, which suggests that it may be important to work preventively in people with varicose veins, for prevention of future VTE or other complications.

## Electronic supplementary material

Below is the link to the electronic supplementary material.

Supplementary material 1 (DOCX 19 kb)
