# Supplementary material for: Self-rated health and venous thromboembolism among middle-aged women: a population-based cohort study
Source: J Thromb Thrombolysis. 2019 Nov 19;49(3):344–51. doi: 10.1007/s11239-019-01995-7 (PMC7145782; doi:10.1007/s11239-019-01995-7)
Supplement: Supplementary file 1 — Supplementary material 1 (DOCX 19 kb) [file 11239_2019_1995_MOESM1_ESM.docx]

Comparison of baseline characteristics between those with incident VTE and those without incident VTE

| Baseline measures | Incident VTE | No incident VTE | P |
| --- | --- | --- | --- |
| Age mean std dev n | 56.57 3.04 319 | 56.29 2.99 5307 | 1.1023 |
| Systolic blood pressure mean (mmHg) std dev n | 132.55 15.96 317 | 132.04 17.10 5295 | 0.6016 |
| Diastolic blood pressure mean (mmHg) std dev n | 85.73 8.76 317 | 85.05 9.25 5297 | 0.2005 |
| Height mean (cm) std dev n | 166.44 6.05 308 | 165.25 5.61 5174 | 0.0003 |
| Weight mean (kg) std dev n | 71.98 13.17 287 | 67.61 11.03 4993 | 0.0000 |
| Body mass index mean std dev n | 29.31 11.95 317 | 28.09 11.37 5284 | 0.0649 |
| Waist mean (cm) std dev  N | 85.14 11.48  288 | 81.66 10.44  4753 | 0.0000 |
| Hip mean (cm) std dev  N | 106.72 9.34  288 | 103.87 8.06  4752 | 0.0000 |
| Waist Hip Ratio mean std dev n | .79 .069 317 | .78 .06 5291 | 0.0004 |
| Smoker  yes Former no | 85(27.4%) 59(18.9%) 168(53.85%) | 1042(19.96%) 1153(22.08%) 3026(57.96%) | 0.007 |
| Alcohol 0 g/w 0-12 g/w >12 g/w | 84(27.54%) 186(60.98%) 35(11.48)% | 1253(24.60%) 3158(62.01%) 682(13.39%) | 0.397 |
| Education 7-9 years 10-12 years > 12 years | 83(26.69%) 128(41.16%) 100(32.15%) | 1214(23.29%) 2117(40.62%) 1881(36.09%) | 0.255 |
| Marital Married Unmarried Divorced Widowed | 233(73.27%) 23(7.23%) 46(14.47%) 16(5.03%) | 3825(72.33%) 359(6.79%) 845(15.98%) 259(4.90%) | 0.904 |
| Physical activity Very low Low  Middle Middle-high Very high | 5(1.59%) 19(6.05%) 155(49.63%) 125(39.81%) 10(3.18%) 0 | 56(1.07%) 222(4.25%) 2686(51.41%) 2032(38.89%) 201(3.85%) 28(.54%) | 0.384 |
| Low activity (Very low & Low)  High activity | 24(7.64%)  290(92.36%) | 278(5.32%) 4947(94.68%) | 0.078 |
| Sugar Daily Sometimes Avoids | 16(5.11%) 214(68.37%) 83(26.52%) | 189(3.59%) 3555(67.50%) 1523(28.92%) | 0.288 |
| Fat in food Much Careful with Avoids | 27(9.03%) 180(60.20%) 92(30.77%) | 328(6.49  %) 3121(61.75%) 1605(31.76%) | 0.229 |
| Dietary fiber in food Low intake  Regularly High intake | 4(1.28%) 212(67.73%) 97(30.99%) | 78(1.49%) 3097(59.17%) 2059(39.34%) | 0.011 |
| Fruit  Eats rarely Eats regularly  Much fruit | 8(2.52%)  106(33.44%)  203(64.04%) | 100(1.90%)  1927(36.52%) 3250(61.57%) | 0.431 |
| Overall diet Less healthy  Healthy | 47(14.78%)  271(85.22%) | 587(11.09%)  4707(88.91%) | 0.043 |
| Self-rated health 1.Very poor 2 3 4 5 6 7.Excellent | 4(1.28%) 6(1.92%) 26(8.31%) 52(16.61%) 79(25.24%) 89(28.43%) 57(18.21%) | 28(0.54%) 110(2.11%) 297(5.69%) 710(13.61%) 1299(24.90%) 1618(31.02%) 1154(22.12%) | 0.090 |
| Self-rated health group  Poor  Good | 88(28.12%) 225(71.88%) | 1145(21.95%) 4071(78,05%) | 0.011 |
| Amount of food Big portions Regularly Small portions | 17(5.86%) 189(65.17%) 84(28.97%) | 363(7.3%) 3086(62.34%) 1501(30.32%) | 0.513 |
| Diabetes Yes  No | 6(1.89%) 311(98.11%) | 102(1.94%) 5158(98.06%) | 0.954 |
| Hypertonia before baseline Yes  No | 15(4.70%) 304(93.390) | 280(5.28%) 5027(94.72%) | 0.655 |
| Varicose veins before baseline Yes No | 12(3.76%) 307(96.24%) | 102(1.92%) 5205(98,08%) | 0.024 |
| Acetylsalicylic Yes No | 2(.63%) 317(99.37%) | 37(.70%) 5270(99.30%) | .883 |
| Knowledge about family history Yes No  Do not know | 39(12.58%) 248(80.00%) 23(7.42%) | 509(9.85%) 4274(82.72%) 384(7.43%) | 0.295 |

P-values were calculated with two-sided Student´s t-test for continuous variables and Chi2-test for dichotomized variables between incident VTE and no incident VTE. Physical activity was dichotomized into Low activity the lower tertile (very low and low) High activity (middle-very high) (Figure 2). Self-rated health was dichotomized into the variable Poor self-rated health, with group 1-4 as poor self-rated health and 5-7 as good self-rated health.
